# Glycosaminoglycan-Mimetic Sulfated Chitosan Promotes Extracellular Matrix Formation and Regulates Inflammation to Alleviate Osteoarthritis

**DOI:** 10.3390/bioengineering13050576

**Published:** 2026-05-19

**Authors:** Xinye Chen, Zirui He, Yuanman Yu, Jing Wang, Changsheng Liu

**Affiliations:** 1 The State Key Laboratory of Bioreactor Engineering, East China University of Science and Technology, Shanghai 200237, China; xycwade@163.com (X.C.); liucs@ecust.edu.cn (C.L.); 2 Key Laboratory for Ultrafine Materials of Ministry of Education, East China University of Science and Technology, Shanghai 200237, China; zirui.he@ecust.edu.cn; 3 Frontiers Science Center for Materiobiology and Dynamic Chemistry, East China University of Science and Technology, Shanghai 200237, China

**Keywords:** polysaccharide, osteoarthritis, cartilage regeneration

## Abstract

Osteoarthritis (OA) is a multifactorial degenerative joint disease characterized by chronic inflammation, progressive cartilage extracellular matrix (ECM) degradation, and impaired joint lubrication, creating a complex pathological microenvironment that remains challenging to treat. In this study, a glycosaminoglycan (GAG)-mimetic sulfated chitosan (SCS) was synthesized via chemical modification of chitosan by grafting sulfonic acid groups, aiming to address these pathological features simultaneously. The therapeutic potential of SCS in OA was systematically evaluated. In vitro results demonstrated that SCS significantly promoted ECM synthesis in chondrocytes. Tribological analysis further revealed that SCS effectively enhanced cartilage lubrication in OA porcine cartilage, as evidenced by a marked reduction in the coefficient of friction, which decreased by 19% under a 5 N load and by 30% under a 10 N load. PCR analysis showed that SCS treatment significantly upregulated chondrogenic-related genes. In addition, SCS exhibited pronounced anti-inflammatory effects by downregulating the expression of inflammatory and catabolic genes. Importantly, in vivo studies demonstrated that SCS effectively preserved cartilage ECM and alleviated synovitis. Collectively, these findings indicate that SCS can simultaneously promote cartilage matrix regeneration, improve lubrication, and suppress inflammation, thereby effectively alleviating OA progression in a complex pathological environment. This study highlights the potential of SCS as a multifunctional GAG-mimetic biomaterial for osteoarthritis therapy.

## 1. Introduction

Osteoarthritis (OA) is one of the most prevalent degenerative joint disorders worldwide, characterized by progressive cartilage degradation, subchondral bone remodeling, synovial inflammation, and loss of joint lubrication [1,2,3,4]. The limited self-repair capacity of articular cartilage means that current clinical treatments, such as nonsteroidal anti-inflammatory drugs and hyaluronic acid injections, primarily focus on symptom relief rather than restoring cartilage structure or function [5,6,7]. Moreover, these therapies rarely address the complex pathological features of OA simultaneously, including chronic inflammation, extracellular matrix (ECM) breakdown, and loss of lubrication. These limitations underscore the urgent need for biomaterials capable of both promoting ECM formation and regulating inflammation to comprehensively restore cartilage homeostasis and function [8,9,10].

Glycosaminoglycans (GAGs), such as chondroitin sulfate and hyaluronic acid, are key components of the ECM [8,11,12]. They play essential roles in maintaining cartilage hydration, mechanical resilience, and boundary lubrication [13,14,15]. Additionally, GAGs are involved in regulating cellular behaviors and inflammatory responses [16]. However, the direct application of natural GAGs is often limited by poor stability, rapid degradation, and high cost [8,17,18]. Consequently, there is an unmet need for biomimetic materials that not only replicate the structural features of GAGs but also actively modulate the OA microenvironment to promote regeneration and reduce inflammation [19,20]. Chitosan, a natural polysaccharide derived from chitin, has been widely explored in biomedical applications due to its biocompatibility, biodegradability, and ease of chemical modification [21,22]. Previous studies have also demonstrated its broad utility in tissue engineering and cartilage repair [13,23]. However, native chitosan lacks the sulfated structure of GAGs, which is crucial for their biological functions [14,19]. To address this limitation, sulfation modification can be introduced to chitosan to mimic the negatively charged sulfate groups present in GAGs, thereby enhancing its bioactivity [24,25].

In this study, we synthesized sulfated chitosan (SCS) by grafting sulfonic acid groups at the 2 N and 6 O positions of chitosan, aiming to construct a GAG-mimetic biomaterial [26]. We hypothesized that SCS could promote cartilage regeneration, improve lubrication, and exert anti-inflammatory effects in OA [27,28,29]. To test this hypothesis, we systematically evaluated the biological effects of SCS on chondrocyte ECM production, gene expression, and inflammatory responses in vitro, as well as its therapeutic efficacy in an OA animal model. Additionally, tribological properties were assessed to determine their role in enhancing joint lubrication. Our findings demonstrate that SCS not only promotes cartilage matrix synthesis and lubrication but also effectively suppresses inflammation, highlighting its potential as a multifunctional therapeutic strategy for osteoarthritis.

## 2. Materials and Methods

### 2.1. Synthesis of Sulfated Chitosan

Sulfated chitosan was synthesized based on previously reported methods with slight modifications [30,31]. Briefly, an anhydrous and oxygen-free reaction system was established under argon protection. The reaction flask was flame-dried and evacuated three times to remove residual moisture. Chitosan (Mw ≈ 300 kDa, 2.5 g) was dissolved in a mixture of formamide (50 mL) and formic acid (2 mL) under stirring at room temperature. In parallel, chlorosulfonic acid (5 mL) was slowly added into N, N-dimethylformamide (DMF, 50 mL) in an ice–salt bath and reacted for 1 h to prepare the sulfating reagent. The sulfating solution was then added dropwise to the chitosan solution, followed by reaction at 45–55 °C for 2 h. The reaction mixture was subsequently poured into anhydrous ethanol (500 mL) to precipitate the product. The precipitate was collected by vacuum filtration, redissolved in ultrapure water, and centrifuged (6000 rpm, 15 min) to remove unreacted chitosan. The purification process, including ethanol reprecipitation, was repeated three times. The resulting solution was adjusted to pH 7.2 using 1 M NaOH, followed by dialysis (MWCO 14 kDa) for 3 days with water refreshed twice daily. Finally, the product was freeze-dried to obtain SCS.

### 2.2. Characterization of Sulfonated Chitosan

The chemical structure of chitosan and sulfated chitosan was characterized by Fourier transform infrared (FTIR) spectroscopy. Briefly, the samples were dried thoroughly before analysis. A small amount of each sample was mixed uniformly with potassium bromide (KBr) powder and then compressed into pellets for measurement. FTIR spectra were recorded using a Fourier transform infrared spectrometer in the range of 4000–400 cm^−1^ with a resolution of 4 cm^−1^ over 32 scans. The obtained spectra were used to identify the characteristic functional groups of SCS, particularly the sulfate-related absorption bands.

### 2.3. Isolation and Culture of Chondrocytes from Rat Articular Cartilage

All animal procedures were approved by the Institutional Animal Care and Use Committee of East China University of Science and Technology. Primary articular chondrocytes were isolated from the femoral condyles and tibial plateaus of 4-week-old Sprague–Dawley rats (jsj-lab, Shanghai, China). Briefly, articular cartilage tissues were carefully dissected, minced into small fragments, and subjected to sequential enzymatic digestion. The tissues were first treated with 0.25% trypsin–EDTA (Sigma-Aldrich, St. Louis, MO, USA) at 37 °C with shaking (200 rpm) for 1 h, followed by further digestion in 0.2% collagenase type II (Worthington) at 37 °C for 3 h. The resulting cell suspension was passed through a 70 μm cell strainer to remove undigested debris and then centrifuged to collect the cells. The cell pellet was washed twice with phosphate-buffered saline (PBS) and subsequently resuspended in DMEM/F12 medium supplemented with 10% fetal bovine serum (Gibco) and 1% penicillin–streptomycin. Cells were cultured at 37 °C in a humidified atmosphere with 5% CO_2_. Chondrocytes between passages 2 and 5 were used for subsequent in vitro experiments.

### 2.4. Cytotoxicity Analysis of SCS

The cytotoxicity of SCS was evaluated using both Live/Dead staining and CCK-8 assay. Briefly, chondrocytes were seeded in 96-well plates at a density of 5 × 10^3^ cells/well and allowed to attach overnight. Cells were then treated with SCS or with culture medium alone as the Ctrl group.

For Live/Dead staining, after 24 h and 72 h of incubation, cells were washed with PBS and stained using the Live/Dead viability/cytotoxicity kit according to the manufacturer’s instructions. Live cells fluoresced green (Calcein-AM), whereas dead cells fluoresced red (EthD-1). Fluorescence images were captured using a confocal microscope, and the percentage of live cells was quantified using ImageJ 1.8.0. For the CCK-8 assay, cells were incubated with 10 µL of CCK-8 solution per well for 2 h at 37 °C. Absorbance at 450 nm was measured using a microplate reader.

### 2.5. Concentration Screening of SCS for Promoting Extracellular Matrix Secretion of Chondrocytes In Vitro

Sulfated chitosan, as a glycosaminoglycan-mimetic polysaccharide, has the potential to promote ECM secretion in chondrocytes. To evaluate this effect, in vitro experiments were performed to investigate the ability of SCS to enhance ECM production and to assess its dose-dependent effects. Chondrocytes were seeded in 24-well plates at a density of 1.0 × 10^4^ cells per well. After cell attachment, the cultures were gently washed with phosphate-buffered saline (PBS) to remove non-adherent and dead cells. The medium was then replaced with fresh culture medium containing different concentrations of SCS (100, 200, 400, 600, 800, 1000, 1200, 1400, 1600, 1800, and 2000 ng/mL), while the control group received medium without SCS. The medium was refreshed every 2 days. After 7 days of culture, the cells were washed with PBS and fixed with 4% paraformaldehyde for 15 min. Subsequently, ECM production was evaluated by histological staining using toluidine blue and Safranin O. The staining was performed for 30 min, followed by rinsing with ultrapure water. Finally, the stained samples were observed under an optical microscope to assess ECM deposition.

### 2.6. Tribological Test

The lubricating effect of SCS on OA cartilage was evaluated through in vitro tribological testing. An OA cartilage model was first established using porcine cartilage via enzymatic degradation. Briefly, fresh porcine cartilage was cut into pieces of approximately 15 mm × 15 mm and digested with 0.5% trypsin (20 mL) at 37 °C for 3 h. After digestion, the samples were rinsed with Hank’s balanced salt solution (HBSS) for 5 min, followed by treatment with HBSS containing 20% fetal bovine serum to terminate the enzymatic reaction. The resulting OA cartilage samples were then stored in HBSS prior to testing. Tribological tests were conducted using a high-frequency reciprocating tribometer (UMT-3, Bruker, Karlsruhe, Germany) under a ball-on-plate configuration. Cartilage specimens with relatively smooth surfaces were fixed onto the testing stage, and reciprocating sliding tests were performed. The experimental parameters were set as follows: a polytetrafluoroethylene (PTFE) ball with a diameter of 8 mm was used as the counterface, with applied loads of 5 and 10 N, a sliding amplitude of 4 mm, a frequency of 1 Hz, and a total test duration of 15 min.

### 2.7. RT-qPCR Analysis

Chondrocytes were seeded in 6-well plates at a density of 5 × 10^4^ cells per well. After IL-1β stimulation for 24 h, cells were treated with SCS for 3 days. The cells were then washed with cold PBS and lysed using TRIzol reagent for total RNA extraction. Briefly, the lysate was mixed with chloroform, followed by centrifugation (12,000 rpm, 4 °C, 15 min) to separate phases. The aqueous phase was collected, and RNA was precipitated with isopropanol, washed with 75% ethanol, and dissolved in DEPC-treated water. RNA concentration and purity were determined using a NanoDrop 2000 spectrophotometer (Thermo Fisher, Waltham, MA, USA). cDNA was synthesized using a reverse transcription kit (TAKARA, Tokyo, Japan) according to the manufacturer’s instructions. qPCR was performed using TB Green Master Mix (TAKARA, Tokyo, Japan) in a 20 μL reaction system on a CFX96 real-time PCR instrument (Thermo Fisher, Waltham, MA, USA). The cycling conditions were 95 °C for 30 s, followed by 40 cycles of 95 °C for 5 s and 60 °C for 30 s. Gapdh was used as the internal reference gene. The sequences of the primers used in qPCR are shown in Table 1.

### 2.8. Cellular Immunofluorescence Staining Analysis

Chondrocytes were seeded in 24-well glass-bottom plates (1.0 × 10^4^ cells/well). After attachment, cells were stimulated with IL-1β for 24 h and then treated with medium with or without SCS for 3 days. Cells were fixed with 4% paraformaldehyde, permeabilized using 0.1% Triton X-100, and blocked with 5% goat serum. Samples were incubated with primary antibody (rabbit anti-mouse ACAN) at 4 °C overnight, followed by incubation with Alexa Fluor 561-conjugated secondary antibody for 1 h. The cytoskeleton and nuclei were stained with phalloidin-FITC and DAPI, respectively. Images were acquired using a confocal laser scanning microscope.

### 2.9. Mouse OA Model

All animal experiments were conducted in accordance with the guidelines of the National Institutes of Health for the Care and Use of Laboratory Animals and were approved by the Institutional Animal Care and Use Committee of East China University of Science and Technology (approval number: ECUST-21041; approval date: 12 March 2021). An OA model was established using 3-month-old mice. To induce OA, destabilization of the medial meniscus (DMM) surgery was performed on the right knee under isoflurane anesthesia. Briefly, the joint was exposed under a surgical microscope, and the medial meniscotibial ligament was carefully transected to create joint instability, leading to OA progression. In the sham group, the joint capsule was opened, and the intra-articular structures were exposed without ligament transection, followed by closure of the incision using the same procedure as in the DMM group. The OA mice were randomly divided into three groups (*n* = 5 per group): (1) Sham group (sham-operated control); (2) Ctrl group, receiving intra-articular injection of 10 μL PBS; and (3) SCS group, receiving intra-articular injection of 10 μL SCS solution (1 mg/mL).

### 2.10. Histological Staining

At 8 weeks post-surgery, mice were sacrificed, and knee joints were harvested and fixed in 4% paraformaldehyde at 4 °C for 72 h. Samples were then washed in PBS for 24 h and decalcified in 10% EDTA solution at 4 °C for 72 h. After decalcification, tissues were rinsed with running water, dehydrated through a graded ethanol series, cleared in xylene, and embedded in paraffin. Serial sections (4 μm) were prepared using a microtome (Leica, Heerbrugg, Switzerland) for subsequent histological staining. Paraffin sections were deparaffinized in xylene and rehydrated through graded ethanol to water. H&E staining: Sections were stained with hematoxylin for 2 min and eosin for 2 min, followed by dehydration, clearing, and mounting. The stained sections were observed under an optical microscope. Safranin O–Fast Green Staining: Sections were deparaffinized and rehydrated as described above. Samples were stained with Fast Green for 5 min and briefly differentiated in 1% acetic acid, followed by staining with Safranin O for 5–10 min. After washing, sections were dehydrated, cleared, and mounted. Histological images were obtained using an optical microscope. In Safranin O–Fast Green staining, proteoglycan-rich cartilage matrix appeared red, while bone and collagenous structures were stained green.

Osteoarthritis severity was assessed using the Osteoarthritis Research Society International (OARSI) scoring system. Briefly, Safranin O–Fast Green-stained sections were evaluated for structural changes in the articular cartilage, including cartilage surface integrity, cellularity, and proteoglycan loss. Scores ranged from 0 (normal cartilage) to 6 (severe cartilage erosion extending to the subchondral bone), with higher scores indicating more severe cartilage degeneration [32].

Synovitis score: Synovial inflammation was evaluated on H&E-stained sections using a semi-quantitative 9-point scoring system based on three parameters: (1) enlargement of the synovial lining layer (0–3 points), (2) density of resident stromal cells (0–3 points), and (3) inflammatory cell infiltrate (0–3 points). The sum of the three parameters gave a total synovitis score ranging from 0 to 9, with 0–1 indicating no synovitis, 2–4 low-grade synovitis, and 5–9 high-grade synovitis. Three representative sections per joint were scored independently by two blinded observers, and the mean value was used for analysis [33].

### 2.11. Statistical Analysis

All data are presented as mean ± standard deviation (SD). Statistical analysis and graphing were performed using GraphPad Prism software (10.1.2). Comparisons among multiple groups were conducted using one-way analysis of variance (one-way ANOVA), while differences between two groups were analyzed using an unpaired two-tailed Student’s *t*-test. Statistical significance was determined based on *p* values, with *p* < 0.05 considered statistically significant. Significance levels are indicated as follows: *p* < 0.05 (*), *p* < 0.01 (**), *p* < 0.005 (***), and *p* < 0.0001 (****), while “ns” indicates no significant difference (*p* ≥ 0.05).

## 3. Results

### 3.1. Characterization of SCS and Screening of Chondrocyte Culture Concentration

**Figure 1:**
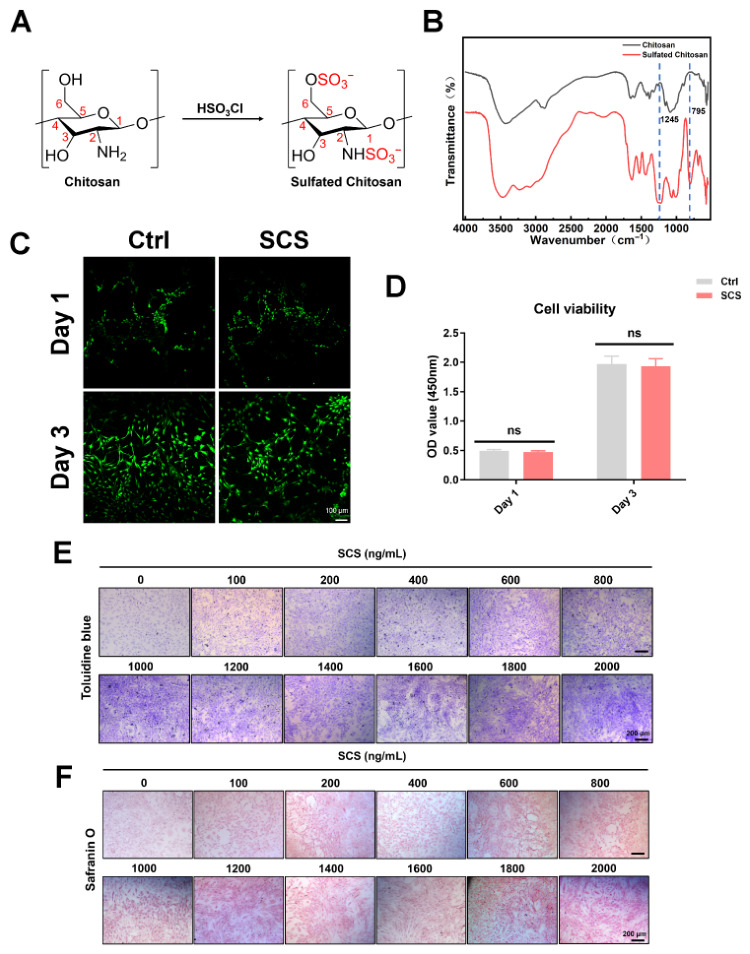
Characterization and Concentration Screening of Sulfated Chitosan. (**A**) Synthesis process of SCS. (**B**) FTIR spectra of Chitosan and Sulfated Chitosan, the blue dash line represents the peak position of sulfonated group. (**C**) Live/Dead fluorescence results of Ctrl and SCS groups on Day 1 and Day 3. (**D**) CCK-8 assay analysis of cell viability on Day 1 and Day 3. ns, not significant. (**E**) Toluidine blue staining of chondrocyte extracellular matrix under the action of different concentrations of SCS. (**F**) Safranin O staining of chondrocyte extracellular matrix under the action of different concentrations of SCS.

In this study, we synthesized SCS through chlorosulfonic acid modification of chitosan and characterized its chemical structure and biological effects. The synthesis process of SCS is illustrated, where sulfonic acid groups are successfully introduced at the 2- and 6-positions of the chitosan backbone (Figure 1A). The successful synthesis of SCS was further confirmed by FTIR spectroscopy (Figure 1B) [27,34]. The FTIR spectrum of unmodified chitosan (black curve) displayed characteristic absorption bands at 1655 cm^−1^ and 1590 cm^−1^, corresponding to amide I (C=O stretching) and amide II (N–H bending) vibrations, respectively, as well as a broad band around 3400 cm^−1^ attributed to O–H and N–H stretching. After sulfation, the FTIR spectrum of SCS (red curve) exhibited two new prominent peaks at 1245 cm^−1^ and 795 cm^−1^, which can be assigned to the asymmetric stretching vibration of the O=S=O group and the stretching vibration of the C–O–S group, respectively. The appearance of these two peaks, together with a slight broadening of the O–H/N–H stretching band, indicates successful introduction of sulfate groups at the 2-N and 6-O positions of chitosan. Notably, the characteristic peaks of native chitosan remained largely unchanged, suggesting that the backbone structure was preserved during the sulfation process. Overall, these FTIR features provide strong evidence for the successful chemical modification of chitosan into SCS.

The cytotoxicity of SCS was evaluated using Live/Dead staining and CCK-8 assay. As shown in Figure 1C, Live/Dead staining revealed that the majority of chondrocytes remained viable after 24 h of incubation with SCS, with cells exhibiting bright green fluorescence and negligible red fluorescence, indicating low cytotoxicity. Compared to the Ctrl group, SCS-treated cells maintained similar morphology and density, suggesting no adverse effects on cell viability (Figure 1C). Consistently, the CCK-8 assay demonstrated that cell viability in the SCS group was 98.7% relative to the Ctrl after 24 h incubation, with no statistically significant difference observed (*p* > 0.05) (Figure 1D). These results collectively indicate that SCS exhibits excellent biocompatibility and does not compromise chondrocyte viability in vitro.

To investigate the potential of SCS in promoting ECM production in chondrocytes, we performed in vitro experiments using various concentrations of SCS. The results of toluidine blue and safranin O staining, used to assess ECM production in chondrocytes exposed to different concentrations of SCS (ranging from 100 ng/mL to 2000 ng/mL), show that the intensity of toluidine blue staining increased with higher concentrations of SCS (Figure 1E). Notably, at 1000 ng/mL, the staining intensity reached a peak, and higher concentrations (e.g., 1200 ng/mL and above) did not significantly further increase the staining intensity, suggesting that 1000 ng/mL was the optimal concentration for promoting ECM production. Similarly, Safranin O staining confirmed this finding. The intensity of the red staining corresponding to proteoglycan-rich cartilage matrix also increased with SCS concentration, reaching a plateau at 1000 ng/mL (Figure 1F). These results indicate that 1000 ng/mL of SCS is the most effective concentration for stimulating ECM synthesis in chondrocytes. The observed concentration-dependent increase in ECM production suggests that SCS enhances the synthesis of key ECM components, such as aggrecan and collagen, in a dose-dependent manner. This effect likely results from the interaction between the sulfated groups in SCS and the chondrocytes, which mimics the structure of natural glycosaminoglycans, thereby promoting ECM production.

### 3.2. Tribological Performance Analysis of SCS

**Figure 2:**
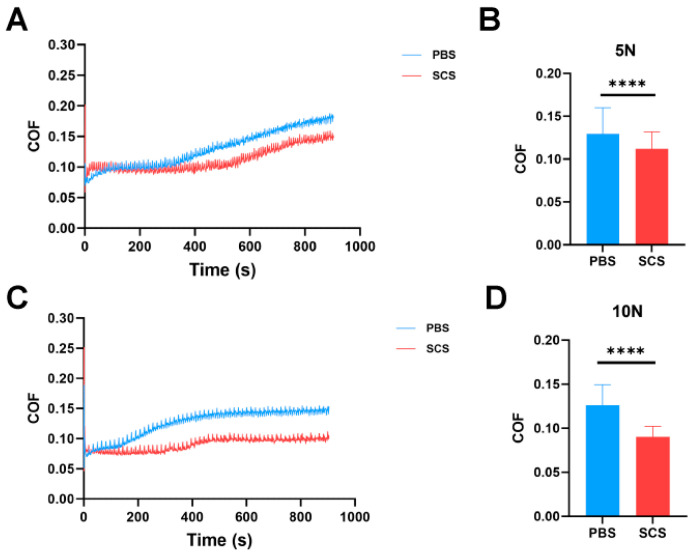
Tribological experiments of SCS. (**A**) Friction curve under 5 N load; (**B**) Quantitative analysis of the average friction coefficient under 5 N load; (**C**) Friction curve under 10 N load; (**D**) Quantitative analysis of the average friction coefficient under 10 N load. Data are shown as means ± SD. Statistical analysis was performed using Student’s *t*-test. **** *p* < 0.001.

In this study, we investigated whether the “biomimetic” properties of SCS allow it to effectively integrate into the joint microenvironment, compensating for or replacing the natural lubricating macromolecules lost from the cartilage surface during osteoarthritis progression [35,36]. We evaluated the lubricating performance of SCS through in vitro tribological tests. To better simulate the lubrication of the OA cartilage surface, porcine articular cartilage treated with trypsin was used as the friction substrate in the tribological experiments. The results of the tribological tests showed that, at 5 N load, there was no significant difference between SCS and the PBS group in the first 300 s of the experiment. However, as the experiment progressed, the coefficient of friction (COF) for SCS became significantly lower than that of the PBS group (Figure 2A). Quantitative analysis revealed that the average COF for the SCS group at 5 N load was reduced by approximately 19% compared to the PBS control group (Figure 2B). At the 10 N load, the COF for SCS remained significantly lower than that of the PBS group throughout the entire test, approximately 0.1, representing a 30% reduction relative to the PBS group (Figure 2C). Quantitative data further confirmed that SCS exhibited significantly lower COF than PBS under both load conditions (Figure 2D). Comparing the two groups at different loads, SCS consistently showed superior lubrication performance, indicating its excellent lubricating effect in joint cartilage [37].

### 3.3. SCS Promotes the Expression of Cartilage Anabolic-Related Genes and Protein Production

**Figure 3:**
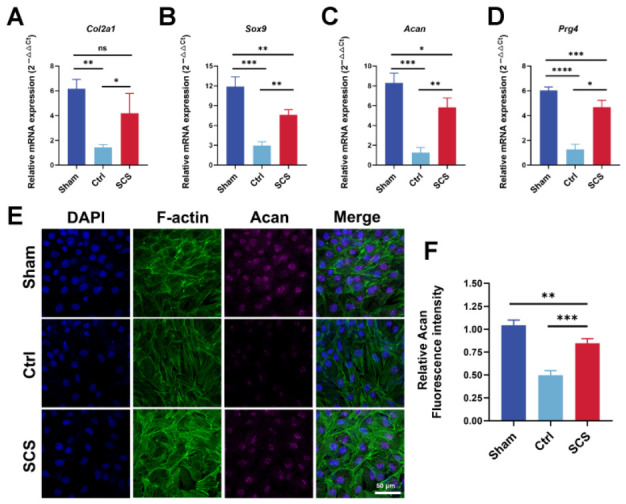
Effect of SCS on cartilage anabolic-related gene expression and protein production. (**A**–**D**) Relative mRNA expression of cartilage-related genes (*Col2a1*, *Sox9*, *Acan*, *Prg4*) in chondrocytes treated with SCS. (**E**) Immunofluorescence staining of Acan in chondrocytes treated with SCS. Cells were stained for Acan (magenta), F-actin (green), and nuclei (DAPI, blue). (**F**) Quantification of Acan fluorescence intensity from the immunofluorescence images. Data are shown as means ± SD. Statistical analysis was performed using one-way ANOVA with Tukey’s post hoc test. * *p* < 0.05, ** *p* < 0.01, *** *p* < 0.005, and **** *p* < 0.001; ns, not significant.

In this study, we investigated the effect of SCS on the expression of key genes involved in cartilage synthesis and homeostasis, including *Col2a1* (collagen type II), *Sox9* (chondrogenic transcription factor), *Acan* (aggrecan), and *Prg4* (lubricin), using RT-qPCR analysis. The results revealed that SCS treatment significantly upregulated the expression of these genes compared to the Ctrl group. Specifically, the mRNA expression of *Col2a1*, *Sox9*, *Acan*, and *Prg4* was increased by approximately 2.2-, 2.5-, 3.8-, and 2.6-fold, respectively, in the SCS-treated group relative to the Ctrl group, suggesting that SCS promotes the synthesis of key cartilage ECM components and enhances chondrocyte anabolic metabolism. (Figure 3A–D).

To further investigate the effect of SCS on protein production, we performed immunofluorescence staining for Acan, a major proteoglycan in cartilage. The fluorescence intensity of Acan was significantly increased in the SCS-treated group compared to the Ctrl group, confirming that SCS enhances the synthesis of aggrecan at the protein level (Figure 3E). Quantitative analysis of the fluorescence intensity revealed that the relative Acan intensity in the SCS group was approximately 1.8-fold higher than in the Ctrl group, indicating enhanced ECM production (Figure 3F). Taken together, these findings demonstrate that SCS promotes the expression of cartilage synthesis-related genes and the production of key cartilage matrix proteins, such as aggrecan. By upregulating the synthesis of anabolic proteins and maintaining cartilage homeostasis, SCS has the potential to restore the balance between matrix synthesis and degradation, making it a promising candidate for the treatment of osteoarthritis and other degenerative cartilage diseases.

### 3.4. SCS Regulates the Expression of Inflammatory and Cartilage Catabolism-Related Genes

**Figure 4:**
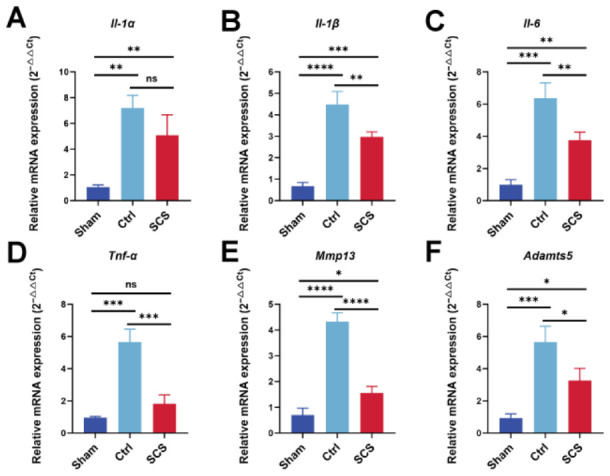
RT–qPCR analysis of inflammation- and cartilage catabolism-related gene expression. (**A**–**F**) Relative mRNA expression levels of *Il-1α* (**A**), *Il-1β* (**B**), *Il-6* (**C**), *Tnf-α* (**D**), *Mmp13* (**E**), and *Adamts5* (**F**) in chondrocytes under different treatments. Data are shown as means ± SD. Statistical analysis was performed using one-way ANOVA with Tukey’s post hoc test. * *p* < 0.05, ** *p* < 0.01, *** *p* < 0.005, and **** *p* < 0.001; ns, not significant.

To further evaluate the regulatory effect of SCS on the inflammatory microenvironment of osteoarthritis, the expression levels of representative inflammatory mediators and cartilage catabolism-related genes were quantitatively analyzed by RT–qPCR. Specifically, six key genes, including *Il-1α*, *Il-1β*, *Il-6*, *Tnf-α*, *Mmp13*, and *Adamts5*, were selected to comprehensively assess inflammation activation and matrix degradation (Figure 4A–F). The results showed that, under inflammatory stimulation, the mRNA expression levels of all inflammatory cytokines in the Ctrl group were significantly upregulated, indicating successful activation of the inflammatory response, which was accompanied by the enhancement of matrix catabolic pathways. In contrast, SCS treatment markedly reduced the expression levels of these genes, with quantitative analysis showing that *Il-1α*, *Il-1β*, and *Il-6* decreased by approximately 32%, 38%, and 45%, respectively, compared to the Ctrl group, and *Tnf-α* was reduced by 60%, reaching a level comparable to the Sham group (Figure 4D).

At the molecular level, *Il-1α*, *Il-1β*, and *Tnf-α*, as key pro-inflammatory cytokines, play critical roles in amplifying inflammatory responses and inducing downstream catabolic signaling during OA progression. Their significant downregulation indicates that SCS effectively suppresses both the initiation and amplification of inflammatory signaling. In addition, the decreased expression of *Il-6*, an important mediator in inflammatory cascades, further demonstrates the ability of SCS to attenuate the progression of inflammation. Importantly, *Mmp13* and *Adamts5*, which are major enzymes responsible for cartilage extracellular matrix degradation, were also significantly downregulated following SCS treatment. *Mmp13* and *Adamts5* expression decreased by approximately 59% and 47%, respectively, relative to the Ctrl group. This suggests that SCS not only inhibits inflammation but also suppresses inflammation-driven matrix degradation processes. By reducing the expression of these catabolic enzymes, SCS may effectively limit the breakdown of collagen and proteoglycans, thereby protecting cartilage structure at the molecular level.

Taken together, these findings demonstrate that SCS exerts a multifaceted regulatory effect within the inflammatory microenvironment by simultaneously modulating inflammatory cytokines and matrix degradation-related genes. Through this dual action, SCS helps restore the balance between anabolic and catabolic processes in cartilage, providing strong evidence for its potential in improving joint homeostasis and preventing cartilage degeneration in OA.

### 3.5. SCS Alleviates Osteoarthritis Progression in Mice

**Figure 5:**
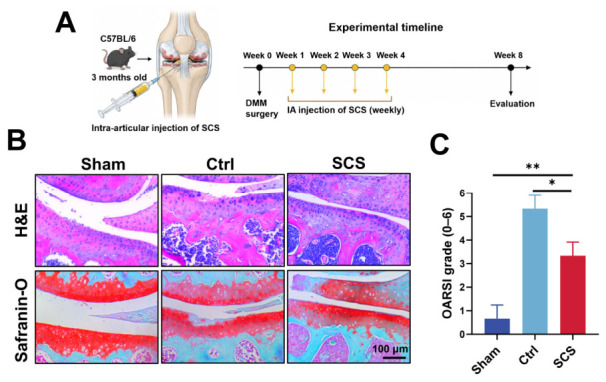
Therapeutic effect of SCS in a mouse OA model. (**A**) Schematic illustration of the experimental timeline, including DMM surgery and intra-articular administration of SCS. (**B**) Representative histological images of knee joints stained with H&E and Safranin O–Fast Green. (**C**) Quantitative analysis of cartilage degeneration based on OARSI scoring. Data are shown as means ± SD. Statistical analysis was performed using one-way ANOVA with Tukey’s post hoc test. * *p* < 0.05, ** *p* < 0.01.

Based on the encouraging in vitro findings demonstrating that SCS can promote cartilage homeostasis and regulate inflammatory responses, we further evaluated its therapeutic efficacy in vivo using a destabilization of the medial meniscus (DMM)-induced OA model in young mice. To investigate the protective effects of SCS, intra-articular injections of SCS were administered once weekly during the first 4 weeks following OA induction. At 8 weeks post-surgery, knee joint tissues were harvested for histological evaluation (Figure 5A).

Hematoxylin and eosin (H&E) staining revealed significant pathological changes in the OA model (Figure 5B). Compared with the Sham group, the Ctrl group exhibited pronounced cartilage degeneration, characterized by surface irregularity, disorganized tissue architecture, and marked thinning of the articular cartilage layer. In contrast, SCS-treated joints showed substantial structural improvement. The cartilage surface appeared smoother, the tissue organization was more preserved, and cartilage thickness was partially restored compared to the untreated OA group, indicating an overall attenuation of cartilage degeneration. Consistent with these observations, Safranin O–Fast Green staining further demonstrated the protective effect of SCS on cartilage ECM. In the Ctrl group, the red staining intensity, representing proteoglycan content, was significantly reduced, reflecting severe ECM loss. However, in the SCS-treated group, the red staining was markedly enhanced, suggesting effective preservation of proteoglycans and improved maintenance of cartilage matrix composition.

Quantitative evaluation using the Osteoarthritis Research Society International (OARSI) scoring system further supported these histological findings (Figure 5C). The SCS-treated group exhibited a significantly lower OARSI score compared with the Ctrl group, indicating reduced cartilage damage and slowed OA progression.

In summary, these results demonstrate that SCS effectively mitigates cartilage degeneration in vivo by preserving ECM integrity and improving tissue structure. Combined with its previously demonstrated anti-inflammatory and pro-anabolic effects, SCS appears to exert a multifaceted therapeutic action, targeting both structural and biochemical aspects of OA pathology. These findings highlight the strong potential of SCS as a promising intra-articular therapeutic agent for the treatment of osteoarthritis.

### 3.6. SCS Regulates Synovial Inflammation in OA Mice

**Figure 6:**
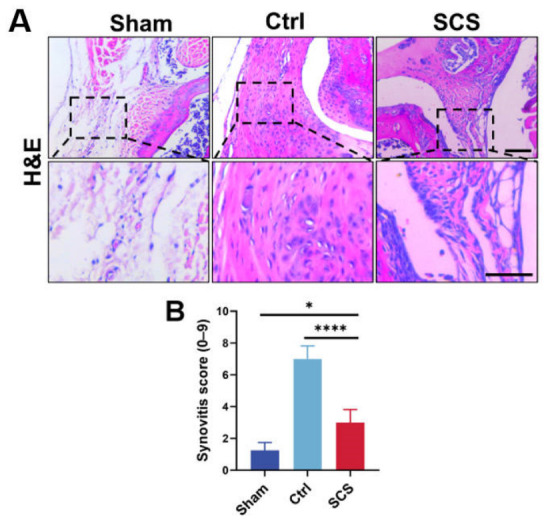
SCS alleviates synovial inflammation in a mouse OA model. (**A**) Representative H&E staining images of synovial tissue; scale bar: 100 μm. (**B**) Quantitative analysis of synovitis scores. Data are shown as means ± SD. Statistical analysis was performed using one-way ANOVA with Tukey’s post hoc test. * *p* < 0.05 and **** *p* < 0.001.

To further evaluate the regulatory effect of SCS on the joint inflammatory microenvironment, synovial inflammation in OA mice was assessed through histological analysis. H&E staining revealed pronounced pathological changes in the synovial tissue of the Ctrl group compared with the Sham group (Figure 6A). Specifically, the synovial membrane exhibited marked thickening, increased lining cell layers, and extensive infiltration of inflammatory cells, indicating a severe inflammatory response. In contrast, SCS treatment via intra-articular injection significantly alleviated these pathological features. The synovial structure in the SCS-treated group was notably improved, with reduced synovial thickness, more organized cellular arrangement, and markedly decreased inflammatory cell infiltration. Higher-magnification images further confirmed these observations: while the Ctrl group displayed typical inflammatory characteristics such as hypercellularity, disorganized stroma, and dense inflammatory cell accumulation, the SCS group maintained a relatively intact synovial architecture with substantially attenuated inflammation. These morphological improvements suggest that SCS effectively mitigates synovial inflammation at the tissue level. Quantitative assessment of synovitis severity further supported these findings. The synovial inflammation score in the SCS-treated group was significantly lower than that in the Ctrl group, with statistical significance, demonstrating the robust anti-inflammatory effect of SCS in vivo (Figure 6B).

These results indicate that SCS effectively suppresses synovial hyperplasia and inflammatory cell infiltration, thereby improving the local joint microenvironment. This tissue-level anti-inflammatory effect is consistent with the previously observed downregulation of pro-inflammatory cytokines at the molecular level, highlighting a coherent mechanism of action. By simultaneously modulating inflammation at both molecular and histological levels, SCS plays a critical role in alleviating joint inflammation and protecting joint structure, further supporting its potential as a promising therapeutic agent for osteoarthritis.

## 4. Discussion

OA is driven by a complex interplay of cartilage matrix degradation, chronic inflammation, and loss of joint lubrication, making single-target therapies often insufficient. In this study, we developed a GAG-mimetic sulfated chitosan to simultaneously address these pathological processes [38]. The introduction of sulfonic acid groups confers SCS with a high density of negative charges, enabling it to structurally and functionally resemble native GAGs within cartilage ECM. By integrating tribological, molecular, and cellular evaluations, our approach provides a systematic assessment of how a single biomaterial can modulate multiple aspects of OA pathology. This biomimetic feature likely underlies its ability to enhance chondrocyte anabolic activity, as reflected by the upregulation of *Col2a1*, *Acan*, *Sox9*, and *Prg4*, as well as increased aggrecan deposition. Mechanistically, the negatively charged sulfate groups may facilitate interactions with growth factors and ECM proteins, thereby promoting matrix assembly and stabilizing the cartilage microenvironment. In addition, the plateau observed at 1000 ng/mL suggests that SCS exerts a dose-dependent effect with a potential saturation of receptor-mediated or matrix-binding interactions, which is consistent with the behavior of other polysaccharide-based biomaterials.

Healthy knee joint articular cartilage exhibits an extremely low coefficient of friction, primarily due to the natural glycosaminoglycans and proteoglycans present in the cartilage surface and synovial fluid [35,39]. These natural macromolecules are rich in anionic groups, particularly sulfate and carboxyl groups. Chitosan, a natural polysaccharide, is known for its good biocompatibility. However, its poor water solubility and relatively limited lubrication performance restrict its application. By chemical modification, sulfonic acid groups are introduced into the molecular structure of chitosan, resulting in sulfated chitosan, which highly mimics the chemical structure, charge properties, and spatial conformation of natural glycosaminoglycans in cartilage, while also exhibiting improved water solubility [34]. In parallel, the significant reduction in friction coefficient observed in tribological tests further supports its functional mimicry of natural lubricating macromolecules [40]. The formation of a hydration lubrication layer mediated by sulfate groups may reduce mechanical wear on cartilage surfaces, thereby breaking the vicious cycle between mechanical stress and biochemical degradation in OA [41].

Beyond its anabolic effects, SCS demonstrated a pronounced capacity to modulate the inflammatory microenvironment and suppress cartilage catabolism. The downregulation of key inflammatory cytokines (*Il-1α*, *Il-1β*, *Il-6*, and *Tnf-α*) indicates that SCS can effectively inhibit the initiation and amplification of inflammatory signaling cascades, which are known to drive OA progression [3,42]. Recent studies have highlighted that immune cells, including macrophages, neutrophils, and mast cells, play critical roles in cartilage degradation and regeneration by secreting pro-inflammatory cytokines, matrix metalloproteinases, and other mediators that alter the cartilage microenvironment [43,44]. Importantly, the concurrent suppression of *Mmp13* and *Adamts5* suggests that SCS interrupts the downstream catabolic pathways responsible for collagen and proteoglycan degradation. Future studies are warranted to directly investigate how SCS interacts with these immune cell populations and modulates their phenotype and function in the OA joint.

The in vivo findings further reinforce the therapeutic relevance of SCS, demonstrating its ability to preserve cartilage structure, reduce OARSI scores, and alleviate synovial inflammation in the DMM-induced OA model. The alignment of molecular, cellular, and tissue-level outcomes highlights the integrative effect of SCS, offering evidence that multifunctional GAG-mimetic polysaccharides can simultaneously modulate biochemical and biomechanical aspects of OA. Notably, the consistency between molecular, cellular, and tissue-level outcomes suggests that SCS exerts a coordinated regulatory effect across multiple biological scales. Future studies focusing on optimizing the molecular structure, delivery strategy, and long-term therapeutic outcomes will be essential to advance SCS toward clinical application. Nevertheless, this study highlights the promise of GAG-mimetic polysaccharides as multifunctional therapeutic platforms for OA treatment, providing a framework for designing biomaterials that address the multifactorial nature of degenerative joint disease.

## 5. Conclusions

In summary, this study developed a glycosaminoglycan-mimetic SCS with multifunctional properties for osteoarthritis treatment. Importantly, SCS simultaneously integrates lubrication restoration, anabolic matrix support, and anti-inflammatory regulation, breaking the vicious cycle between mechanical stress and biochemical degradation in osteoarthritis. By regulating both cartilage homeostasis and the joint inflammatory microenvironment, SCS significantly alleviated OA progression in a mouse model. However, the long-term efficacy and potential systemic effects of SCS remain to be further investigated. These findings highlight the potential of SCS as a promising biomimetic therapeutic strategy for osteoarthritis.

## Figures and Tables

**Table 1:** RT-qPCR primer sequences.

Target Gene	Forward Primer Sequence (5′–3′)	Reverse Primer Sequence (5′–3′)
*Gapdh*	TGACCACAGTCCATGCCATC	GACGGACACATTGGGGGTAG
*Col2a1*	TGCAGAATGGGCAGAGGTAT	ATCTGGGCTGCAAAGTTTCCT
*Sox9*	AGCACTCCGGGCAATCT	GTGTAGACGGGTTGTTCCCA
*Acan*	GACCTGTGTGAGATCGACCA	GTTGGTTTGGACGCCACTTC
*Prg4*	TACTTCTTCAAGAGAGGTGGCA	ATAAGCCATGCAATGGGAG
*Il-1α*	AAGACAAGCCTGTGTTGCTG	TCCCAGAAGAAAATGAGGTC
*Il-1β*	CACCTTCTTTTCCTTCATCTTTG	GTCGTTGCTTGTCTCTCCTTG
*Tnfα*	GTGATCGGTCCCAACAAGGA	TTGGTGGTTTGCTACGACG
*Mmp3*	GGGAAGCTGGACTCGAACACT	TGAGCAGCAACCAGGAATA
*Mmp13*	ACCCAGCCCTATCCCTTGAT	GGCCCAGAATTTTCTCCCTCT
*Adamts5*	TGCCCACCTAACGGCAAAT	TCCTGTTTCCATCCTGGCAC

## Data Availability

Data are contained within this article.

## Abbreviations

The following abbreviations are used in this manuscript:

OA	Osteoarthritis
SCS	Sulfated Chitosan
ECM	Extracellular Matrix
RT-qPCR	Real-time quantitative polymerase chain reaction
